# Supplementary material for: A shared mechanism for Bacteroidota protein transport and gliding motility
Source: Nat Commun. 2025 Nov 20;16:10217. doi: 10.1038/s41467-025-65003-8 (PMC12635249; doi:10.1038/s41467-025-65003-8)
Supplement: Supplementary file 1 — Supplementary Information [file 41467_2025_65003_MOESM1_ESM.pdf]

Supplementary Information

A shared mechanism for Bacteroidota protein transport and gliding motility

Xiaolong Liu[1]*, Marieta Avramova[1], Justin C. Deme[2], Rachel L. Jones[1], Camilla A.K. Lundgren[1], Susan M. Lea[2,3]*, and Ben C. Berks[1]*

[1] Department of Biochemistry, University of Oxford, Oxford, United Kingdom.

[2] Center for Structural Biology, Center for Cancer Research, National Cancer Institute, Frederick, Maryland, United States of America.

[3] Structural Biology, St Jude Children's Research Hospital, Memphis, Tennessee, United States of America.

* Corresponding authors. Email ben.berks@bioch.ox.ac.uk, susan.lea@stjude.org, xiaolong.liu@bioch.ox.ac.uk

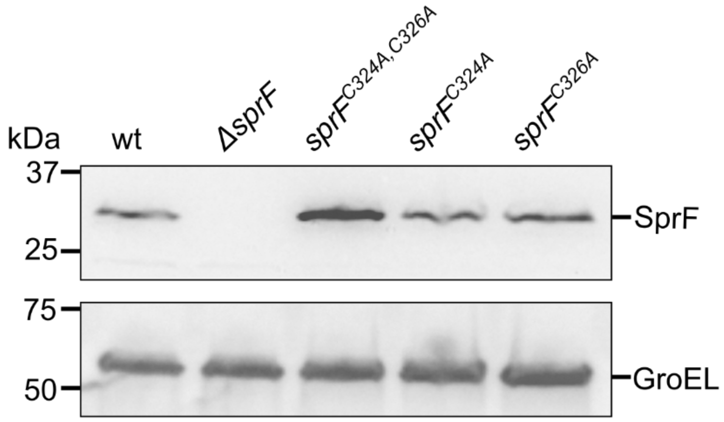

Supplementary Figure 1 | Substitutions of the SprF cysteines residues do not negatively affect the expression or stability of SprF. Whole cells were analysed by immunoblotting with SprF antibodies. GroEL serves as loading control. Similar results were obtained from 3 biological repeats.

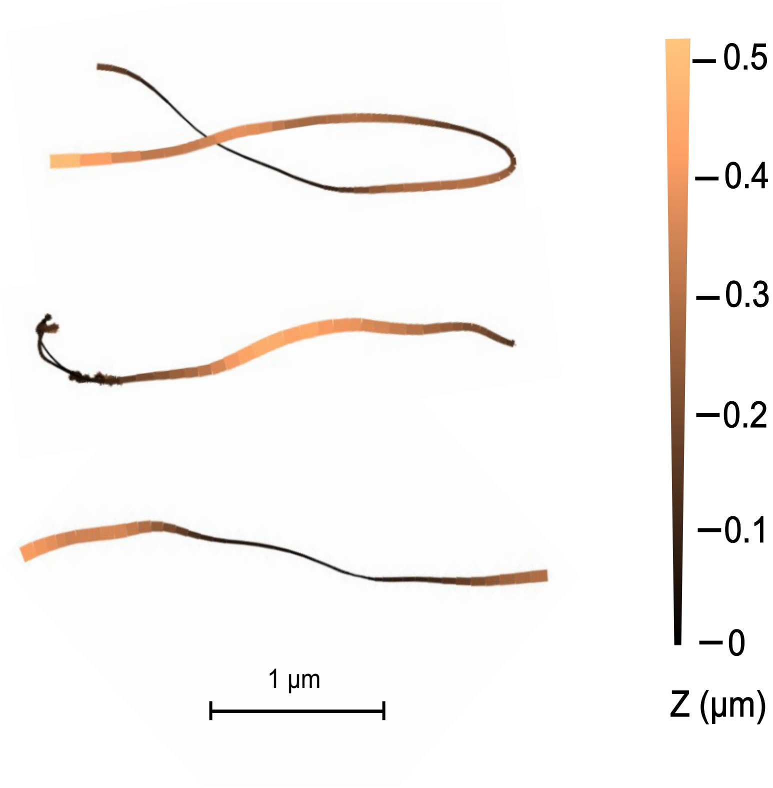

Supplementary Figure 2 | 3D single particle tracking of fluorescently-tagged SprB adhesin molecules in SprF cysteine-substituted variants. The motions of single fluorophore-labelled HaloTag-SprB adhesin molecules were imaged in 3D in agarose-immobilised cells of strains with single and double cysteine substitutions in SprF. The short, apparently directional trajectories present in these cells were extracted and the single molecule localizations in successive frames plotted in xy projection with the relative z position represented by variations in colour and line width as specified in the colour bar. Three representative trajectories are shown. 100 out of 100 such trajectories (from three biological repeats of the three cysteine mutants) where the handedness could be determined showed left-handed helicity consistent with the movement being on the gliding tracks.

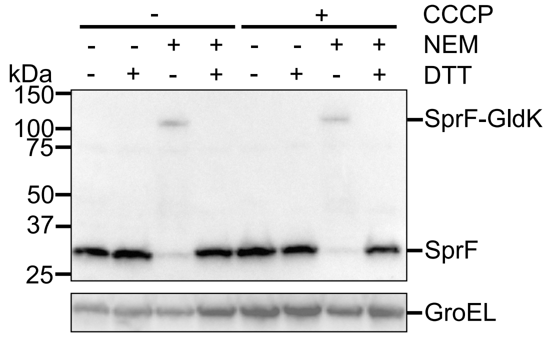

Supplementary Figure 3 | The disulfide adduct involving SprF is stable to removal of the protonmotive force. Immunoblots of whole wild type cells. Where indicated the cells were treated with 10 μM of the protonophore carbonyl cyanide *m*-chlorophenyl hydrazone (CCCP) for 20 min before analysis. Where indicated cells were treated with NEM. At the SDS-PAGE step samples were run under either non-reducing (- DTT) or reducing (+ DTT) conditions. GroEL serves as a loading control. Similar results were obtained from three biological repeats.

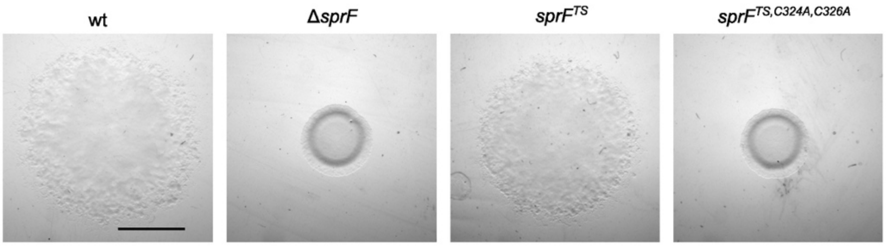

Supplementary Figure 4 | Insertion of a Twin-Strep-tag into Loop 9 (fourth periplasmic loop) of SprF does not affect gliding mobility on agar. Strain *sprF*^{TS} has a Twin-Strep-coding sequence inserted after codon *glu219*. Spreading (gliding) morphology on agar. Scale bar, 5mm. Similar results were obtained from 3 biological repeats.

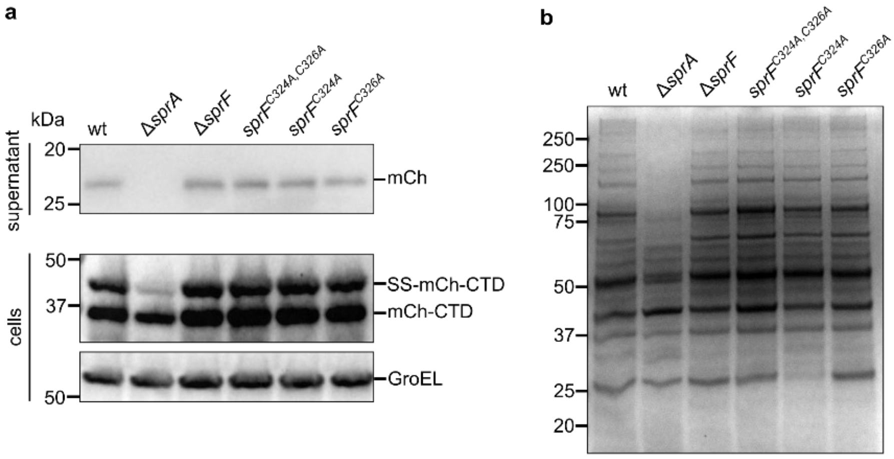

Supplementary Figure 5 | The GldK-SprF disulfide linkage is not required for the secretion of T9SS Type A substrates. Similar results were obtained from three biological repeats.

a, Analysis of the secretion of a model Type A substrate protein comprising a fusion between a signal sequence (SS), mCherry (mCh), and a T9SS-targeting C-terminal domain (CTD). Whole cell and supernatant samples were analyzed by anti-mCherry immunoblotting. The successively processed forms of the fusion proteins are indicated to the right of the blots. The Δ*sprA* strain is missing the T9SS translocon. GroEL serves as a loading control.

b, Secretome analysis of SprF C-tail cysteine variants. The Δ*sprA* strain is missing the T9SS translocon.

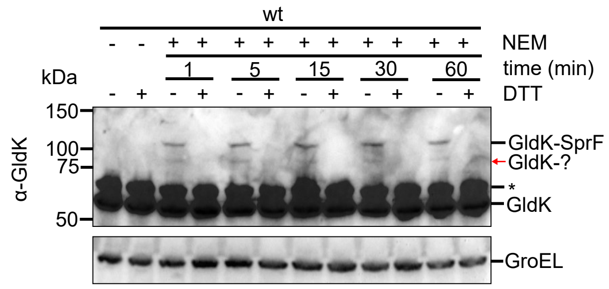

Supplementary Figure 6 │GldK forms disulfide linkages with SprF and another protein. Wild type cells were subject to whole cell immunoblotting with the specified antisera. Where indicated the cells were pre-treated with NEM for the specified time. At the SDS-PAGE step samples were run under either non-reducing (- DTT) or reducing (+ DTT) conditions. GroEL serves as a loading control. The red arrow indicates a low intensity disulfide-linked adduct band formed by GldK that is additional to the adduct with SprF. *, non-specific background band.

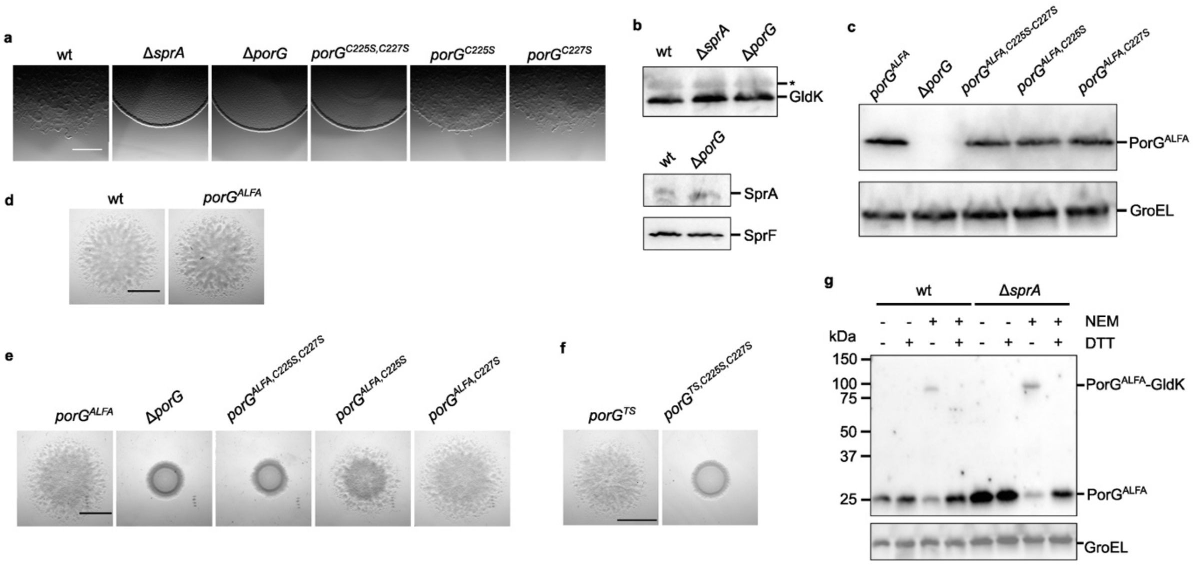

Supplementary Figure 7 | Characterization of *F. johnsoniae* PorG.

a, Spreading (gliding) morphology of non-tagged PorG cysteine variants at 6 h. Scale bar, 1 mm.

b, *F. johnsoniae* PorG is not required for the expression and stability of the key T9SS and gliding components GldK, SprA, and SprF. Whole cell immunoblotting using antibodies against the indicated proteins. *, non-specific band.

c-e, Characterization of strains with an ALFA-tag (**c-e**, *porG*^{ALFA}) or Twin-Strep-tag (**f**, *porG*^{TS}) inserted into Loop 3 (the second extracellular loop) of *F. johnsoniae* PorG after Glu219.

c, Whole cells immunoblotting using ALFA-tag antibodies. GroEL serves as loading control.

d-f, Spreading (gliding) morphology on agar. Scale bar, 5mm.

g, Disulfide bond formation between PorG and GldK does not require T9SS activity. Strains with the indicated mutations were subject to whole cell immunoblotting with ALFA-tag antibodies. Where indicated the cells were pre-treated with NEM. At the SDS-PAGE step samples were run under either non-reducing (- DTT) or reducing (+ DTT) conditions. GroEL serves as a loading control. Δ*sprA*, strain missing the T9SS translocon.

a-g, Similar results were obtained from 3 biological repeats. wt, wild type.

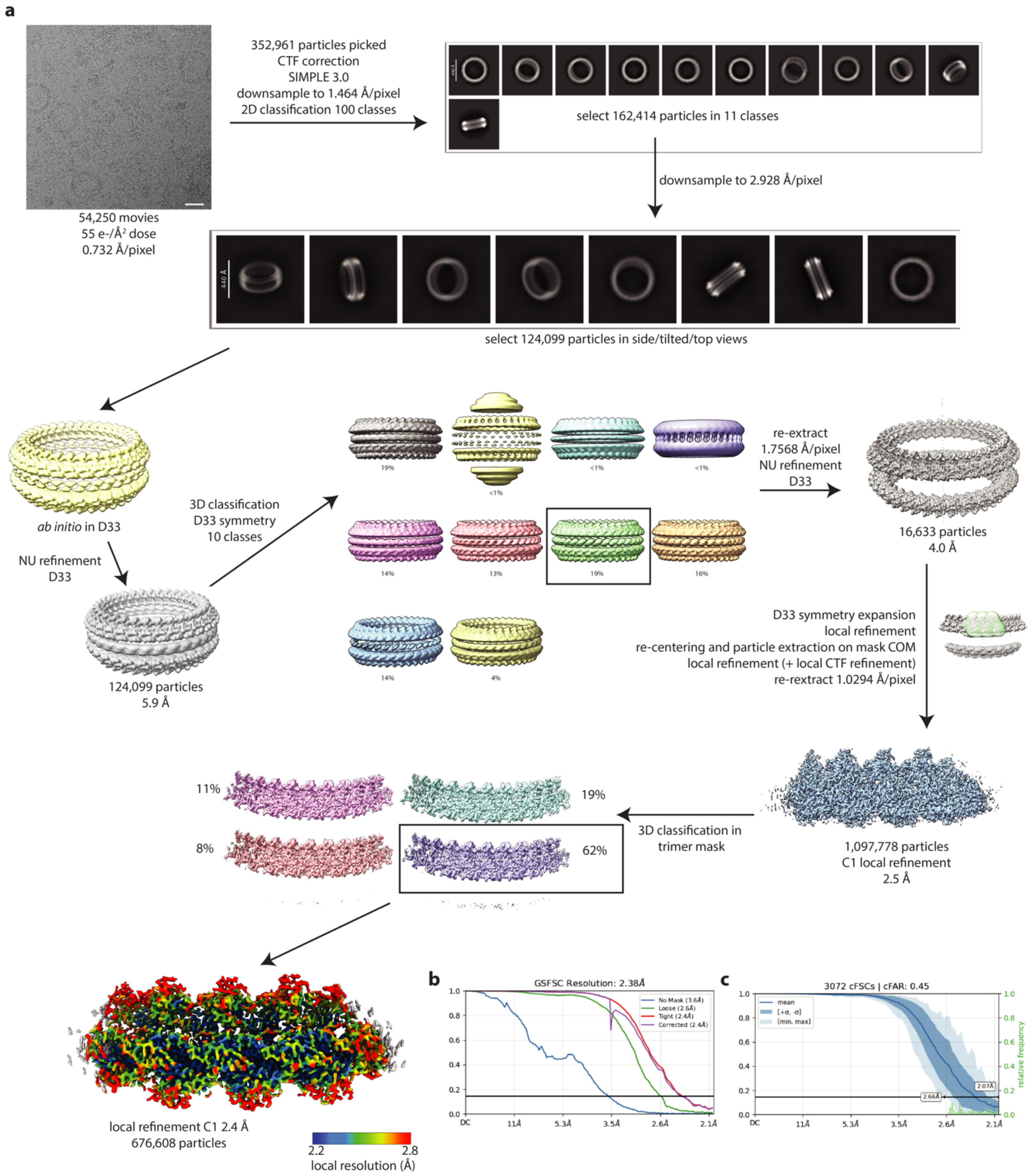

Supplementary Figure 8 | Cryo-EM Workflow for the *P. gingivalis* PorKN complex structure.

a, Image processing workflow, including the local resolution estimate of the volume. Micrograph scale bar is 300 Å.

b, Gold-standard Fourier Shell Correlation (FSC) curves used for global resolution estimation. **c**, Conical FSC (cFSC) plot assessing directional signal content.

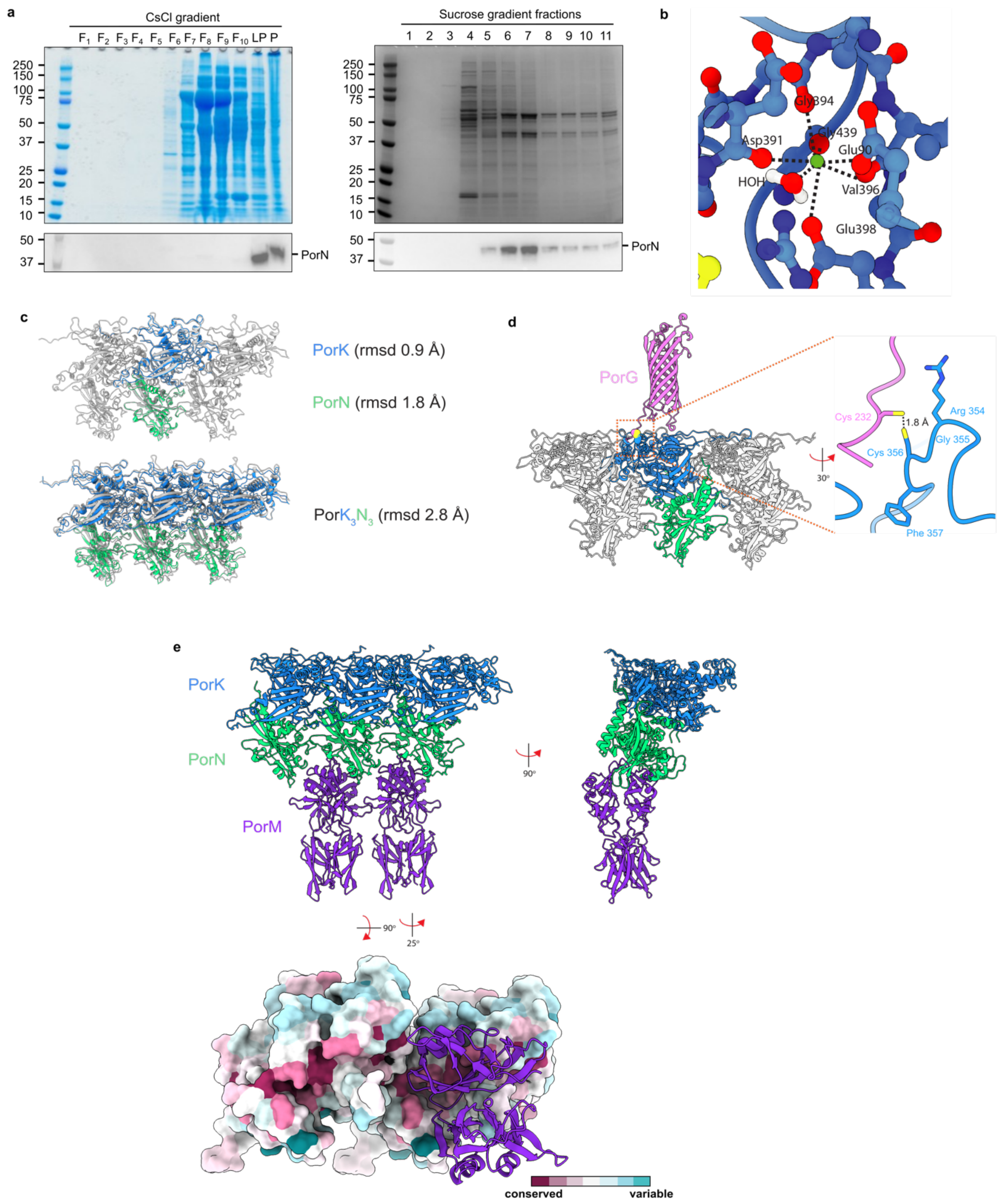

Supplementary Figure 9 | Further information on the *P. gingivalis* PorKN complex.

a, Purification of the PorKN complex for structural analysis. The successive CsCl and sucrose density gradient steps of an exemplar preparation are shown. Coomassie Blue stained SDS-PAGE gels with corresponding anti-PorN immunoblots below. Fractions (F) are numbered from the tops of the gradients. P, pellet; LP, loose pellet. Fraction LP from the CsCl was loaded onto the sucrose gradient from which fractions F6 and F7 were pooled and used for structure determination.

b, The Ca^{2+} ion binding site in PorK with the metal ion (green) and co-ordinating ligands shown as atomic spheres.

c, AF3 reliably models the PorKN oligomer. AF3 models (coloured cartoons) of the isolated PorK and PorN subunits (Top) or of the complex between three PorKN units (Bottom) are superpositioned on a three PorKN unit section of the experimental structure (grey cartoons) with the indicated RMSDs. See also Supplementary Data 1.

d, AF3 model in cartoons representation for the complex formed between PorG and the PorKN ring. The model contains one copy of PorG and three copies of the PorKN unit. No protein-protein protein contacts are predicted beyond the intersubunit disulfide linkage. See also Supplementary Data 2.

e, AF3 model for the complex formed between the distal ends of the PorM motor arms and the PorKN ring. The model contains three copies of the PorKN unit and four copies of the D3-D4 fragment of PorM. Note that each PorM arm is a homodimer. (Top) Two views of the model in cartoons representation. (Bottom) The model viewed from the periplasm but with the PorKN units shown in spacefilling representation and coloured by sequence conservation. One of the PorM homodimer arms has been removed to allow the PorM interaction surface on PorKN to be inspected. The remaining PorM homodimer arm is shown in cartoons representation with the upper PorM subunit forming the potential specific interaction with PorKN. See also Supplementary Data 3.

Supplementary Table 1. Cryo-EM data collection, refinement and validation statistics

	PorKN (PDB 9MZU) (EMD-48769)
**Data collection and processing**	
Magnification	165,000
Voltage (kV)	300
Electron exposure (e–/Å^2)	55.0
Defocus range (μm)	-2.0 to -0.1
Pixel size (Å)	1.0294
Symmetry imposed	C1
Initial particle images (no.)	352,961
Final particle images (no.)	676,608
Map resolution (Å)	2.4
FSC threshold	0.143
Map resolution range (Å)	2.3-38.4
**Refinement**	
Initial model used (PDB code)	none
Model resolution (Å)	2.6
FSC threshold	0.5
Map sharpening *B* factor (Å^2)	-74.3
Model composition	
Non-hydrogen atoms	17511
Protein residues	2094
Ligands	RAM:6<br>GCU:6<br>FUC:6<br>GLC:6<br>CA:3<br>MAN:12
B factors (Å^2)	
Protein	46.1
Ligand	65.2
R.m.s. deviations	
Bond lengths (Å)	0.003
Bond angles (°)	0.495
Validation	
MolProbity score	2.0
Clashscore	12.0
Poor rotamers (%)	2.0
Ramachandran plot	
Favored (%)	96.7
Allowed (%)	3.2
Disallowed (%)	0.1
CC (mask)	0.86
