# Supplementary material for: A shared mechanism for Bacteroidota protein transport and gliding motility
Source: Nat Commun. 2025 Nov 20;16:10217. doi: 10.1038/s41467-025-65003-8 (PMC12635249; doi:10.1038/s41467-025-65003-8)
Supplement: Supplementary file 2 — Description of Additional Supplementary Files [file 41467_2025_65003_MOESM2_ESM.pdf]

Description of Additional Supplementary Files:

Supplementary Data 1. AlphaFold prediction for the structure of a $PorK_3PorN_3$ section of the *P. gingivalis* Hub complex. See also Supplementary Fig. 9c.

Supplementary Data 2. AlphaFold prediction for the structure of a $PorK_3PorN_3$ section of the P. gingivalis Hub complex disulfide-bonded to a PorG molecule. See also Supplementary Fig. 9d.

Supplementary Data 3. AlphaFold prediction for the structure of a $PorK_3PorN_3$ section of the *P. gingivalis* Hub complex bound to four copies of the D3-D4 domains of PorM. See also Supplementary Fig. 9e.

Supplementary Data 4. AlphaFold prediction for the structure of a *F. johnsoniae* $GldJ_3GldK_3GldN_3$ complex. See also Fig. 6b.

Supplementary Data 5. Table of strains.

Supplementary Data 6. Table of plasmids.

Supplementary Data 7. Table of primers.

Supplementary Movie 1. Mobility of fluorophore-tagged SprB adhesin molecules in *F. johnsoniae* cells with mutations in *sprF*. Raw images were recorded with continuous exposure at 33 fps using epifluorescence microscopy. Playback is in real time. The area of the field of view is 32.7 x 32.7 µm.

Supplementary Movie 2. Mobility of fluorophore-tagged SprB adhesin molecules in *F. johnsoniae* cells with mutations in *gldK*. Raw images were recorded with continuous exposure at 33 fps using epifluorescence microscopy. Playback is in real time. The area of the field of view is 32.7 x 32.7 µm.

Supplementary Movie 3. Mobility of fluorophore-tagged SprB adhesin molecules in *F. johnsoniae* cells with mutations in *porG*. Raw images were recorded with continuous exposure at 33 fps using epifluorescence microscopy. Playback is in real time. The area of the field of view is 32.7 x 32.7 µm.

Supplementary Movie 4. Single particle tracking of a fluorophore-tagged SprB adhesin molecule in a *F. johnsoniae* $porG^{C225S,C227S}$ mutant cell showing mid-cell reversals. Raw images were recorded with continuous exposure at 33 fps using epifluorescence microscopy. Overlaid on the image is the real time progression of the super-resolution fit of the adhesin trajectory, coloured by time. Playback is in real time. The scale bar represents 1 µm.

Supplementary Movie 5. Animation of the *P. gingivalis* PorKN Hub complex cryoEM volume determined in this work placed within the cryoET volume assigned to this complex in intact cells in EMD-24227. The cryoEM volumes are coloured blue for PorK and green for PorN.

Supplementary Movie 6. Animation of a PorKN pair and their immediate neighbours within the *P. gingivalis* Hub complex model. At the start of the animation the subunits are viewed from the ring exterior with the outer membrane-facing side of the structure at Top. The PorK subunit is coloured blue for PorK and the PorN subunit green. The position of the PorG-reactive Cys 356 on PorK is shown in yellow.
