# Supplementary material for: A shared mechanism for Bacteroidota protein transport and gliding motility
Source: Nat Commun. 2025 Nov 20;16:10217. doi: 10.1038/s41467-025-65003-8 (PMC12635249; doi:10.1038/s41467-025-65003-8)
Supplement: Supplementary file 16 — Reporting Summary [file 41467_2025_65003_MOESM16_ESM.pdf]

nature portfolio

Corresponding author(s): Ben C. Berks, Susan M. Lea, and Xiaolong Liu

Last updated by author(s): 12th September 2025

Reporting Summary

Nature Portfolio wishes to improve the reproducibility of the work that we publish. This form provides structure for consistency and transparency in reporting. For further information on Nature Portfolio policies, see our Editorial Policies and the Editorial Policy Checklist.

Statistics

For all statistical analyses, confirm that the following items are present in the figure legend, table legend, main text, or Methods section.

n/a	Confirmed	
☐	☒	The exact sample size (n) for each experimental group/condition, given as a discrete number and unit of measurement
☒	☐	A statement on whether measurements were taken from distinct samples or whether the same sample was measured repeatedly
☒	☐	The statistical test(s) used AND whether they are one- or two-sided<br>*Only common tests should be described solely by name; describe more complex techniques in the Methods section.*
☒	☐	A description of all covariates tested
☒	☐	A description of any assumptions or corrections, such as tests of normality and adjustment for multiple comparisons
☒	☐	A full description of the statistical parameters including central tendency (e.g. means) or other basic estimates (e.g. regression coefficient) AND variation (e.g. standard deviation) or associated estimates of uncertainty (e.g. confidence intervals)
☒	☐	For null hypothesis testing, the test statistic (e.g. F, t, r) with confidence intervals, effect sizes, degrees of freedom and P value noted<br>*Give P values as exact values whenever suitable.*
☒	☐	For Bayesian analysis, information on the choice of priors and Markov chain Monte Carlo settings
☒	☐	For hierarchical and complex designs, identification of the appropriate level for tests and full reporting of outcomes
☒	☐	Estimates of effect sizes (e.g. Cohen's d, Pearson's r), indicating how they were calculated

Our web collection on statistics for biologists contains articles on many of the points above.

Software and code

Policy information about availability of computer code

Data collection	
Data analysis	SIMPLE 3.0<br>PHENIX 1.21<br>COOT 0.9.8.95<br>MolProbity 4.5.2<br>UCSF ChimeraX-1.9<br>cryoSPARC v4.5.3<br>ThunderSTORM 1.3<br>Trackpy 0.6.2<br>Custom sotware: https://github.com/notdrjones/Single-Particle-Tracking-for-Disulfide-Bond-Paper

For manuscripts utilizing custom algorithms or software that are central to the research but not yet described in published literature, software must be made available to editors and reviewers. We strongly encourage code deposition in a community repository (e.g. GitHub). See the Nature Portfolio guidelines for submitting code & software for further information.

Data

Policy information about availability of data

All manuscripts must include a data availability statement. This statement should provide the following information, where applicable:

- Accession codes, unique identifiers, or web links for publicly available datasets
- A description of any restrictions on data availability
- For clinical datasets or third party data, please ensure that the statement adheres to our policy

Electron Microscopy DataBank (EMDB): EMD-48769
Protein Data Bank (PDB): PDB 9MZU
Gel and immunoblot source data are published alongside this paper.

Research involving human participants, their data, or biological material

Policy information about studies with human participants or human data. See also policy information about sex, gender (identity/presentation), and sexual orientation and race, ethnicity and racism.

Reporting on sex and gender	N/A
Reporting on race, ethnicity, or other socially relevant groupings	N/A
Population characteristics	N/A
Recruitment	N/A
Ethics oversight	N/A

Note that full information on the approval of the study protocol must also be provided in the manuscript.

Field-specific reporting

Please select the one below that is the best fit for your research. If you are not sure, read the appropriate sections before making your selection.

☒ Life sciences ☐ Behavioural & social sciences ☐ Ecological, evolutionary & environmental sciences

For a reference copy of the document with all sections, see nature.com/documents/nr-reporting-summary-flat.pdf

Life sciences study design

All studies must disclose on these points even when the disclosure is negative.

Sample size	EM sample sizes were determined by available electron microscope time and density of particles on the grids. All biological experiments were carried out in triplicate.
Data exclusions	EM: Particles were excluded from final analysis using pre-established classification methods within the software packages described. Briefly, 2D classification was used to exclude classes that represented contaminations, while 3D classification was used to remove particles that didn't belong to clearly defined structural elements. Details are given in the manuscript.<br>Other data: no data were excluded.
Replication	The number of repeats for each experiment are given in the Figure legends and in all cases the data were successfully reproduced.
Randomization	No randomisation was performed as samples were targeted for analysis.
Blinding	No blinding was performed. For single particle tracking all track selection was automated.

Reporting for specific materials, systems and methods

We require information from authors about some types of materials, experimental systems and methods used in many studies. Here, indicate whether each material, system or method listed is relevant to your study. If you are not sure if a list item applies to your research, read the appropriate section before selecting a response.

Materials & experimental systems

n/a	Involved in the study
☐	☒ Antibodies
☒	☐ Eukaryotic cell lines
☒	☐ Palaeontology and archaeology
☒	☐ Animals and other organisms
☒	☐ Clinical data
☒	☐ Dual use research of concern
☒	☐ Plants

Methods

n/a	Involved in the study
☒	☐ ChIP-seq
☒	☐ Flow cytometry
☒	☐ MRI-based neuroimaging

Antibodies

Antibodies used

anti-HaloTag (G921A, Promega), anti-GroEL (G6532 Merck), anti-mCherry (ab167453, Abcam), anti-ALFA-Tag (N1582, Synaptic Systems GmbH), anti-Strep-tag (34850 Qiagen), anti-mouse IgG peroxidase conjugate (A4416 Merck), and anti-rabbit IgG peroxidase conjugate (31462 Pierce). Antisera against SprF (Kulkarni et al. 2019), GldK (Shrivastava et al. 2013), and SprA (Nelson et al. 2007) were provided by Mark McBride (University of Wisconsin-Milwaukee). Antibodies against GldJ and PorN were raised in rabbits against the peptides specified in the text. Antibodies were used at the following dilutions: anti-Strep-tag 1:2000; anti-SprF 1:2500; anti-GldJ, anti-GldK, anti-PorN, anti SprA, anti-ALFA-tag, anti-HaloTag, anti-mCherry, and all secondary antibodies, 1:3000; anti-GroEL 1:50000.

Validation

Validated by immunoblotting strains expressing the target antigen versus strains not expressing the target antigen. Anti-GroEL cannot be validated in this way but is used only as a a loading control as in multiple previous studies, fractionates to the cytoplasm, has the correct molecular mass, and has been validated by the commercial supplier.

Plants

Seed stocks

N/A

Novel plant genotypes

N/A

Authentication

N/A
